# Supplementary material for: High dielectric barium titanate porous scaffold for efficient Li metal cycling in anode-free cells
Source: Nat Commun. 2021 Nov 11;12:6536. doi: 10.1038/s41467-021-26859-8 (PMC8585873; doi:10.1038/s41467-021-26859-8)
Supplement: Supplementary file 1 — Supplementary Information [file 41467_2021_26859_MOESM1_ESM.pdf]

Supporting Information

High dielectric barium titanate porous scaffold for efficient Li metal cycling in anode-free cells

Chao Wang[1], Ming Liu[1], Michel Thijs[2], Frans G.B. Ooms[1], Swapna Ganapathy[1] and Marnix Wagemaker[1*]

[1] Section Storage of Electrochemical Energy, Radiation Science and Technology, Faculty of Applied Sciences, Delft University of Technology. E-mail: m.wagemaker@tudelft.nl

[2] Neutron & Positron Methods for Materials, Radiation Science and Technology, Faculty of Applied Sciences, Delft University of Technology.

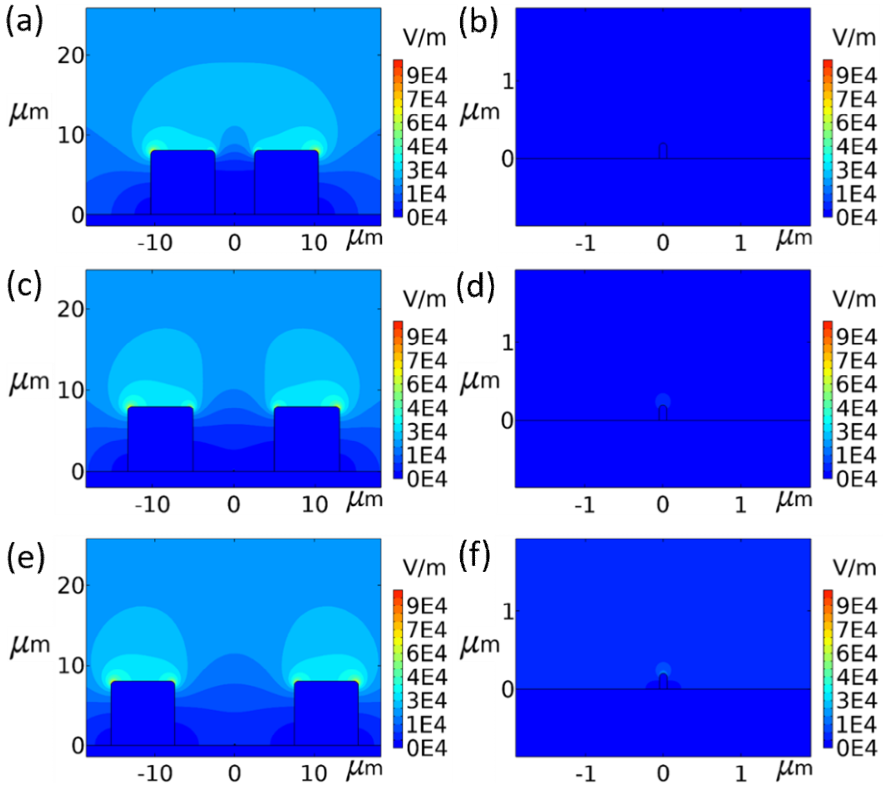

Supplementary Figure 1 Schematic diagram of the electric field distribution of $BaTiO_3$ (BTO) coated Cu with different block gaps. (a, b) with distance of 5 μm between blocks and zoomed-in figure, (c, d) with distance of 10 μm between blocks and zoomed-in figure, (e, f) with distance of 15 μm between blocks and zoomed-in figure.

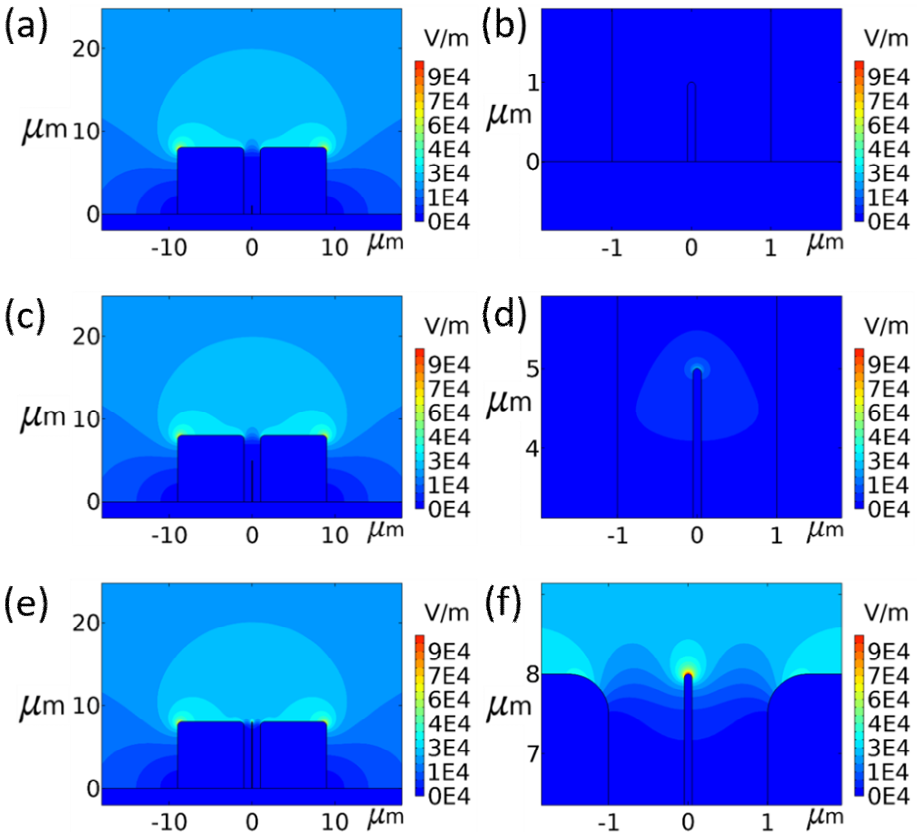

Supplementary Figure 2 Schematic diagram of the electric field distribution of $BaTiO_3$ (BTO) coated Cu with different dendrite length. (a, b) with a dendrite length of 1 μm and zoomed-in figure, (c, d) with a dendrite length of 5 μm and zoomed-in figure, (e, f) with a dendrite length of 8 μm and zoomed-in figure.

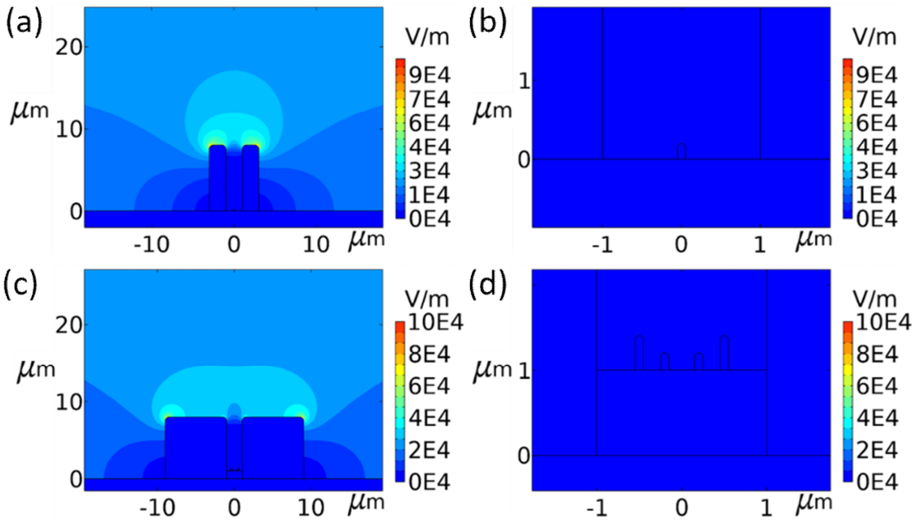

Supplementary Figure 3 Schematic diagram of the electric field distribution of $BaTiO_3$ (BTO) coated Cu (a, b) with a block width of 2 μm and zoomed-in figure, (c, d) with multiple dendrites and zoomed-in figure.

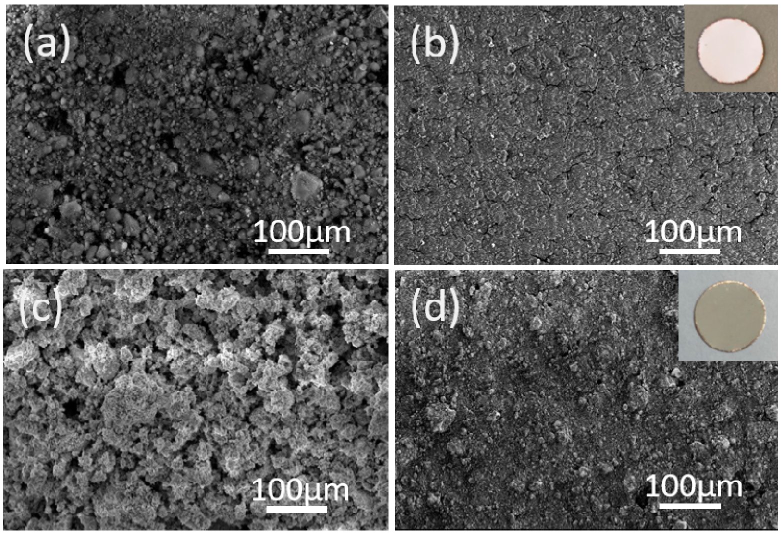

Supplementary Figure 4 ball-milled starting materials and their coatings on copper. (a, b) AO and AO coated on Cu, (c, d) BTO and BTO coated on Cu. Insets are the digital image of the electrode.

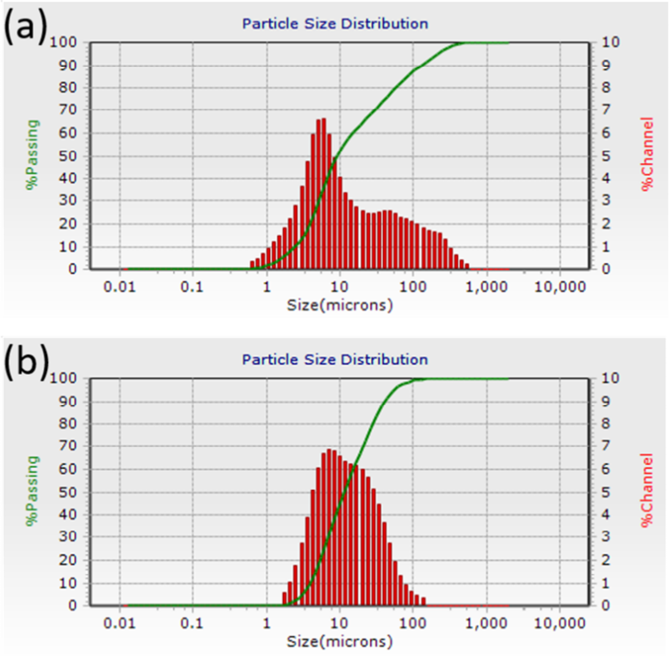

Supplementary Figure 5 Particle size distribution of (a) BTO and (b) AO as obtained using the Dynamic Light Scattering technique.

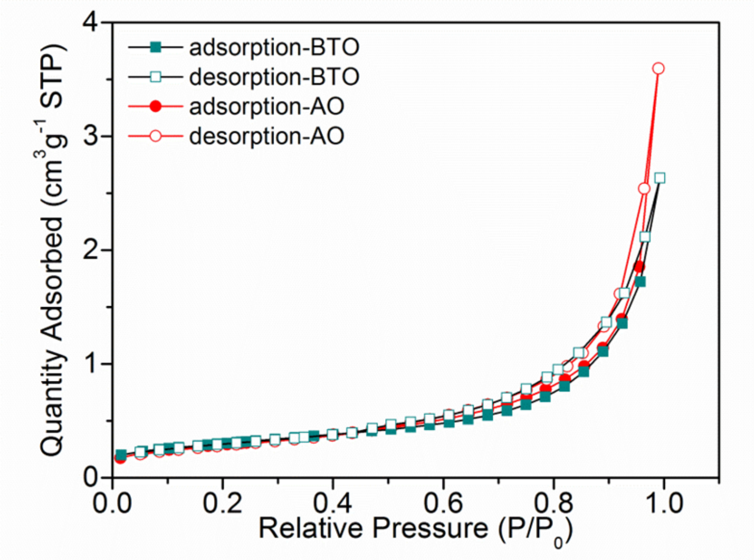

Supplementary Figure 6 Nitrogen adsorption/desorption isotherms at 77 K of BTO and AO materials.

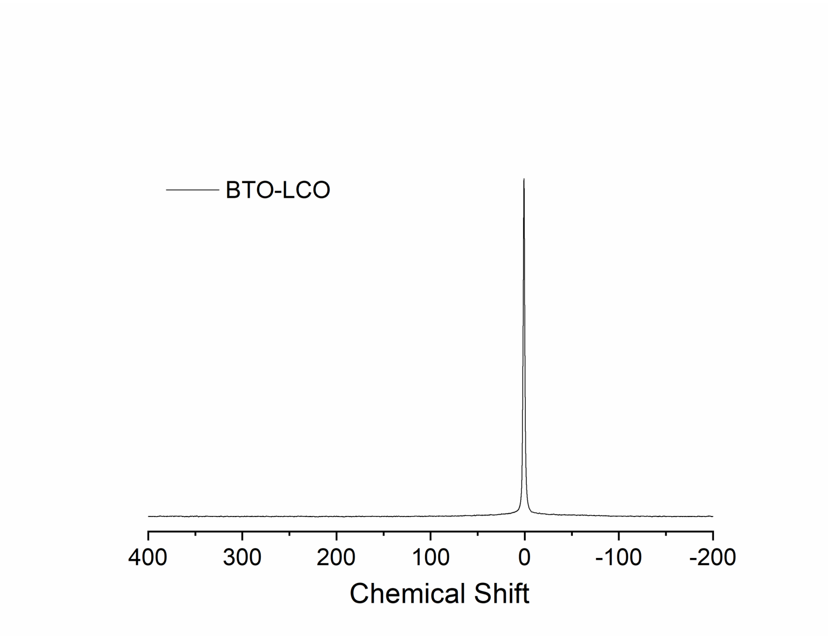

Supplementary Figure 7 Static ^{7}Li NMR spectrum measured with a wide spectral window of a fresh BTO-LCO battery.

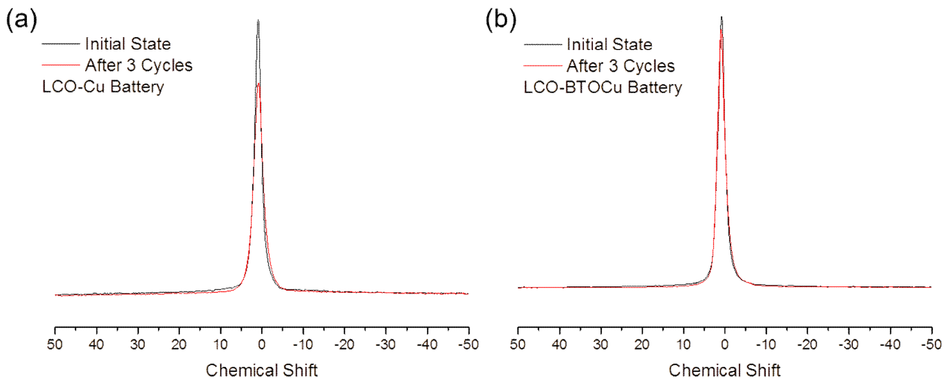

Supplementary Figure 8 Anode-free (a) Cu and (b) BTO scaffold on Cu versus a $LiCoO_2$ cathode with a 1M $LiPF_6$ EC/DMC electrolyte cycled at 0.2 mA cm^{-2} to 1 mA cm^{-2} charge capacity and discharge to 2.5 V cut-off 1D ^7Li solid-state NMR spectra at selected conditions (50 to -50 ppm).

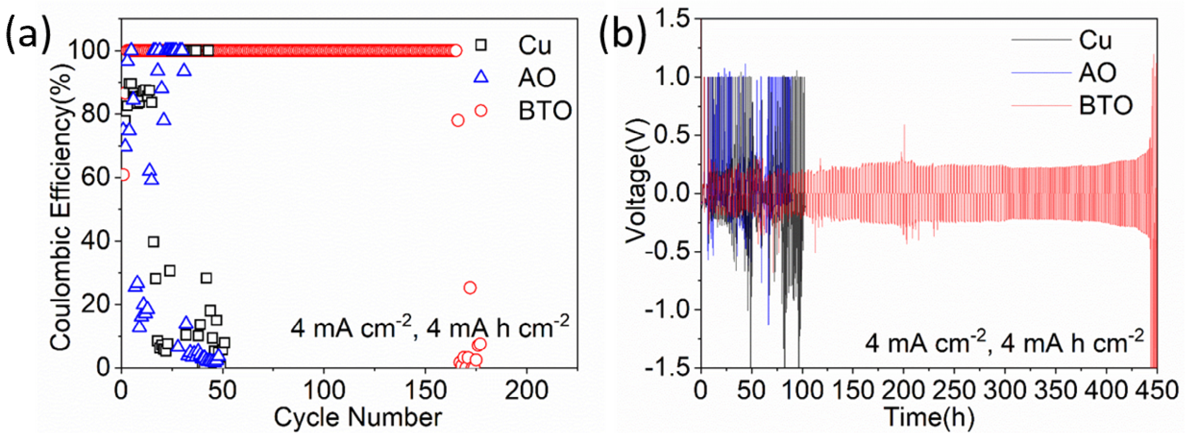

Supplementary Figure 9 (a) Lithium Coulombic efficiencies at a fixed areal capacity of 4 mA h cm^{-2} at 4 mA cm^{-2} and (b) the corresponding voltage files.

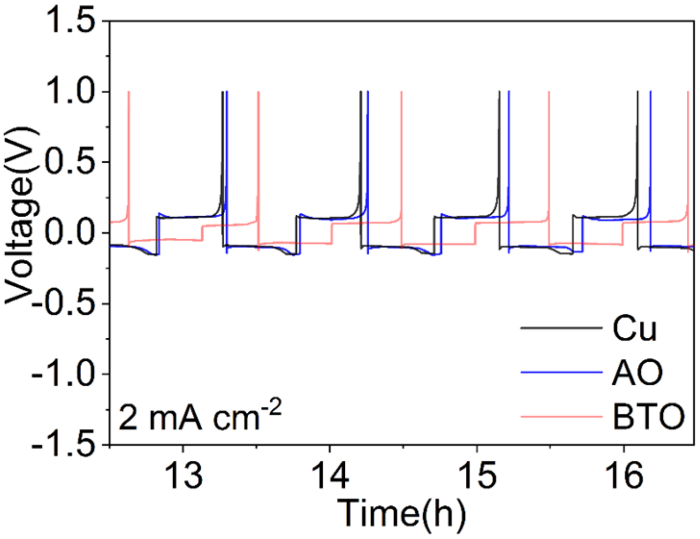

Supplementary Figure 10 The evolution of the voltage during the anode-free half-cell cycling with a voltage limited stripping at 1 V vs Li/Li^+ at 2 mA cm^{-2}.

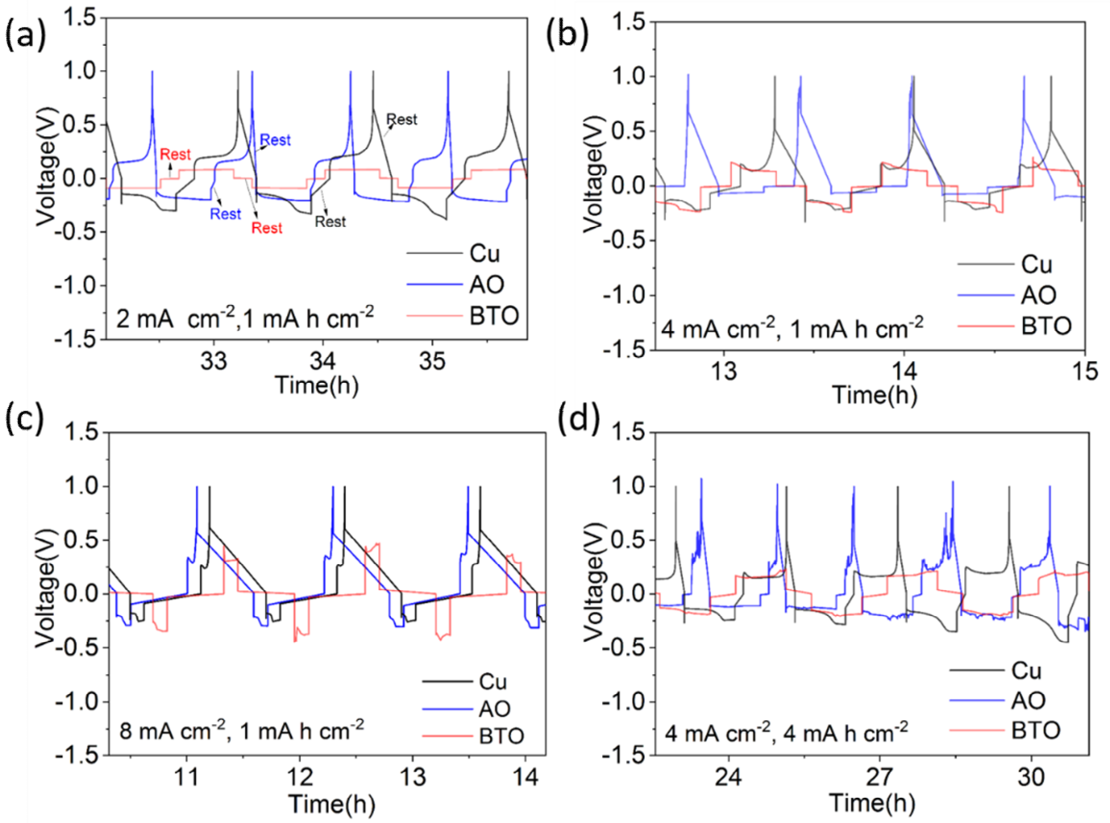

Supplementary Figure 11 Detailed voltage profiles of the anode-free Cu, AO and BTO scaffolds vs a Li metal anode. Evolution of the voltage during Li plating/stripping for the bare Cu and the AO and BTO scaffolds at different current densities (a) 2 mA cm^{-2}, (b) 4 mA cm^{-2}, (c) 8 mA cm^{-2} with a fixed areal capacity of 1 mA h cm^{-2}, and (d) an increased areal capacity of 4 mA h cm^{-2} at a current density of 4 mA cm^{-2}. Note that each constant current step is followed by a 2 to 30 minutes rest, as indicated in (a).

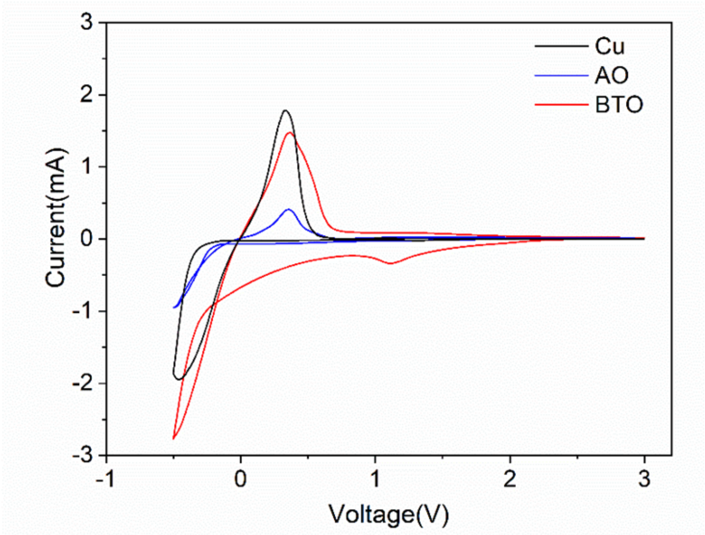

Supplementary Figure 12 Cyclic voltammetry performance of different electrodes with a scan rate of 1mV s^{-1} in the range of -0.5 V to 3 V.

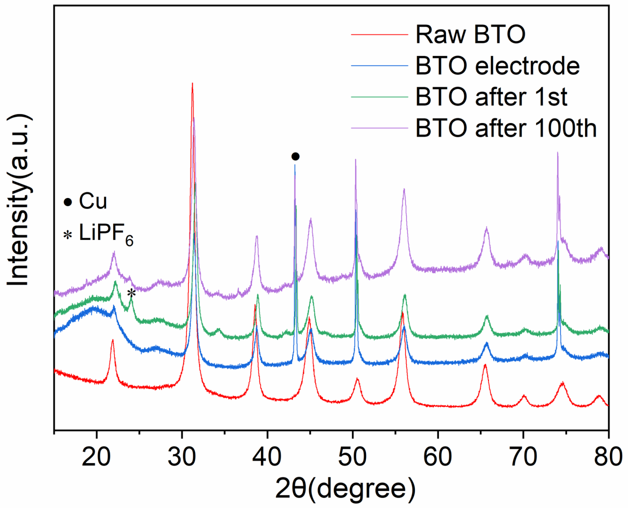

Supplementary Figure 13 Ex situ postmortem XRD patterns of BTO raw material and BTO electrodes at the 1st and 100th cycles.
